# Monocyte Chemoattractant Protein-1 Secreted by Decidual Stromal Cells Inhibits NK Cells Cytotoxicity by Up-Regulating Expression of SOCS3

**DOI:** 10.1371/journal.pone.0041869

**Published:** 2012-07-27

**Authors:** Xiaofei Xu, Qingjie Wang, Biping Deng, Huayang Wang, Zhaogang Dong, Xun Qu, Beihua Kong

**Affiliations:** 1 Department of Obstetrics and Gynecology, Qilu Hospital, Shandong University, Jinan, Shandong, China; 2 Institute of Basic Medical Sciences, Qilu Hospital, Shandong University, Jinan, Shandong, China; 3 Department of Clinical Laboratory, Qilu Hospital, Shandong University, Jinan, Shandong, China; Hannover Medical University (MHH), Germany

## Abstract

**Background:**

Decidual stromal cells (DSCs) are of particular importance due to their pleiotropic functions during pregnancy. Although previous research has demonstrated that DSCs participated in the regulation of immune cells during pregnancy, the crosstalk between DSCs and NK cells has not been fully elucidated. To address this issue, we investigated the effect of DSCs on perforin expression in CD56^+^ NK cells and explored the underlying mechanism.

**Methodology/Principal Findings:**

Flow cytometry analysis showed perforin production in NK cells was attenuated by DSC media, and it was further suppressed by media from DSCs pretreated with lipopolysaccharide (LPS). However, the expression of granzyme A and apoptosis of NK cells were not influenced by DSC media. ELISA assays to detect cytokine production indicated that monocyte chemoattractant protein-1 (MCP-1) in the supernatant of DSCs conditioned culture significantly increased after LPS stimulation. The inhibitory effect of DSC media on perforin was abolished by the administration of anti-MCP-1 neutralizing antibody. Notably, reduced perforin expression attenuated the cytotoxic potential of CD56^+^NK cells to K562 cells. Moreover, Suppressor of cytokine signaling 3 (SOCS3) expression in NK cells was enhanced by treatment with MCP-1, as measured by RT-PCR and western blot. Interestingly, MCP-1-induced perforin expression was partly abolished by the siRNA induced SOCS3 knockdown. Western blot analysis suggested that both NF-κB and ERK/MAPKs pathway were involved in the LPS-induced upregulation of MCP-1 in DSCs.

**Conclusions/Significance:**

Our results demonstrate that LPS induces upregulation of MCP-1 in DSCs, which may play a critical role in inhibiting the cytotoxicity of NK cells partly by promoting SOCS3 expression. These findings suggest that the crosstalk between DSCs and NK cells may be crucial to maintain pregnancy homeostasis.

## Introduction

Successful pregnancy is not only an immunologically paradoxical, but also an exactly regulated event. The maternal immune system must be activated, which contributes to the maintenance of host defense against microbial pathogens to avoid adverse outcomes [1]. Furthermore, the embryo expresses paternal allo-antigens and establishes a successful symbiosis with the mother, which requires that the maternal immune system must be suppressed [2]. Therefore, there must be a sophisticated balance between inflammatory and immune inhibitory responses in the maternal immune system. However, little is known about the underlying mechanisms.

Decidual tissues to which a semi-allograft is directly exposed are considered to be crucial for feto-maternal immune tolerance during pregnancy [3]. Decidual stromal cells (DSCs) that differentiate from fibroblast-like precursor cells are the main cellular component of the decidual tissue. Except for their traditionally nutritious and supportive roles in pregnancy [4], growing evidences suggest that DSCs may be involved in immunomodulation, such as antigen phagocytosis [5] and presentation [6], through direct cell-to-cell interactions [7]. Moreover, these versatile cells express a wide variety of chemokines and cytokines that orchestrate the immunological context of the feto-maternal interface [8], [9]. However, whether these soluble factors participate in feto-maternal tolerance during pregnancy has not been fully elucidated.

One of the most distinct features of pregnancy is that a large amount of leukocytes are recruited into the decidual tissue from the peripheral blood, and they are then precisely programmed to ignore the invasion and development of the fetal semi-allograft [2]. Among these cells, NK cells have attracted considerable attention because they consist of the majority of the decidual immune cells [10], [11]. DSCs make a substantial contribution to the modulation of the peripheral immune cells that infiltrate into the deciduas [7], [12]. It has been reported that TGF-β released from the DSCs promotes the conversion of CD16^+^ NK cells in the peripheral blood into decidual CD16^−^ NK cells [8]. However, the effects of DSCs on the cytotoxicity of NK cells and the related regulatory mechanisms have not been fully understood. In the current study, we explore whether soluble factors secreted from DSCs participate in the regulation of NK cells.

The production of cytokines by DSCs is affected by the local decidual microenvironment. Several endogenous inflammatory factors, such as IL-1β [13] and TNF-α [14], may regulate the expression of cytokines in DSCs. Moreover, pattern recognition receptors, or TLRs, that can recognize exogenous microorganisms are found at maternal-fetal interface. The activation of TLRs triggers an array of signaling pathways and influences cytokine and chemokine production, which in turn regulate the following immune response [15]. Each TLR is distinct in its specificity. DSCs constitutively express TLR4 [16], which can specifically bind to LPS produced by the predominant gram-negative bacteria in the genital tract [17], suggesting that TLR4 on DSCs may regulate the effect of DSCs on NK cells.

In the present study, we observed that DSC-conditioned media suppressed perforin expression in CD56^+^ NK cells, which was further inhibited by DSC-conditioned media from LPS-stimulated cells (DSC-LPS media). However, granzyme A (GrzA) expression was not influenced by either of these media. In addition, no difference was found in the effect exerted by either of these media on NK cell apoptosis, suggesting that both media inhibit perforin production directly. Next, we confirmed that it was MCP-1 present in the media mediated the interaction between DSCs and NK cells. MCP-1 inhibited perforin production by promoting suppressor of cytokine signaling (SOCS3) expression in NK cells. Furthermore, we found LPS triggered the NF-κB and ERK/MAPK pathways in DSCs, which in turn up-regulated MCP-1 production and finally enhanced the inhibition of perforin expression of NK cells by DSCs.

## Results

### Identification of human first-trimester DSCs

**Figure 1:**
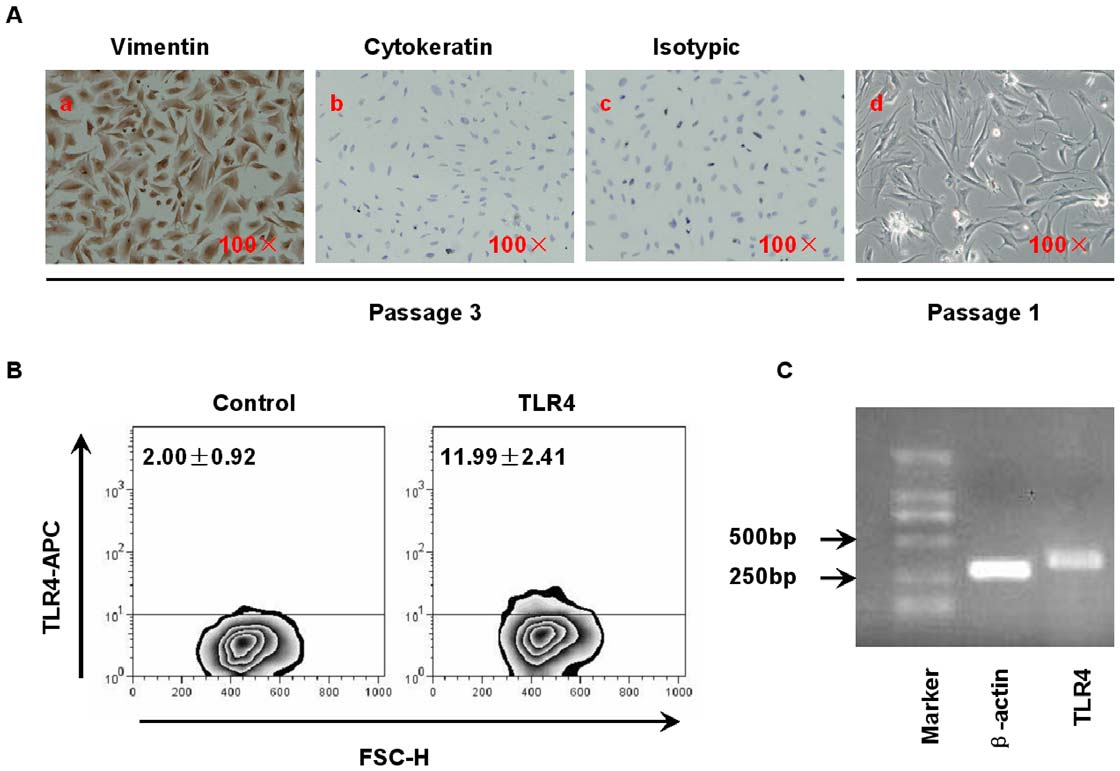
Identification of human first-trimester DSCs. (A) Purity assessment of DSCs by immunocytochemistry. Positive staining is indicated in brown color. (A.a) and (A.b), respectively represent passage-3 DSCs stained with anti-vimentin and anti-cytokeratin antibodies (×100), (A.c) is the isotypic control (×100). (A.d) Freshly isolated DSCs without any treatment (×100). This is a representative photograph from 5 identically conducted experiments. (B) Examination of TLR4 expressed on DSCs. After 48 hours of culture in complete RPMI medium, DSCs were harvested as described in *Materials and Methods*, stained with an APC-conjugated anti-TLR4 mAb or an isotypic mAb and analyzed by flow cytometry. The data in the figure represent the percentage of DSCs expressing TLR4.The figure is a representative plot based on five independent experiments, each of which was repeated three times. The mean±SD was used to represent the percentage of the population. (C) Detection of TLR4 transcription in DSCs. The DSCs were dissociated, and the otal RNA was extracted for RT-PCR. This is a representative experiment from 5 independent experiments using separate samples.

To characterize the purity of cultured DSCs after 3–4 passages, we determined the expression of vimentin and cytokeratin in these cells by immunocytochemistry. Fig. 1A.d indicates that freshly isolated cells were contaminated by other cells. After 3 passages, almost all of the cells showed vimentin immunoreactivity (brown, Fig. 1A.a) and had similar morphology to cells at passage 1, and there were no cytokeratin- positive cells (Fig. 1A.b). Furthermore, flow cytometry demonstrated that the percentage of CD45^+^ cells was <1% (data not shown), which suggested that the cell population was free of leukocytes.

To investigate whether DSCs still expressed TLR4 after 3–4 passages at levels similar to those in vivo or in freshly isolated cells, we examined the expression of TLR4 by flow cytometry and RT-PCR. The cultured cells were incubated with APC-conjugated anti-TLR4 mAb for 30 minutes at room temperature and then analyzed. Fig. 1B showed that although at a lower level 11.99%±2.41%, DSCs still constitutively expressed TLR4 after 3–4 passages. Fig. 1C indicated the transcriptional level of TLR4 by RT-PCR.

### The effects of DSC-conditioned and DSCs-LPS media on perforin expression, apoptosis and GrzA expression in human CD56^+^ NK cells

**Figure 2:**
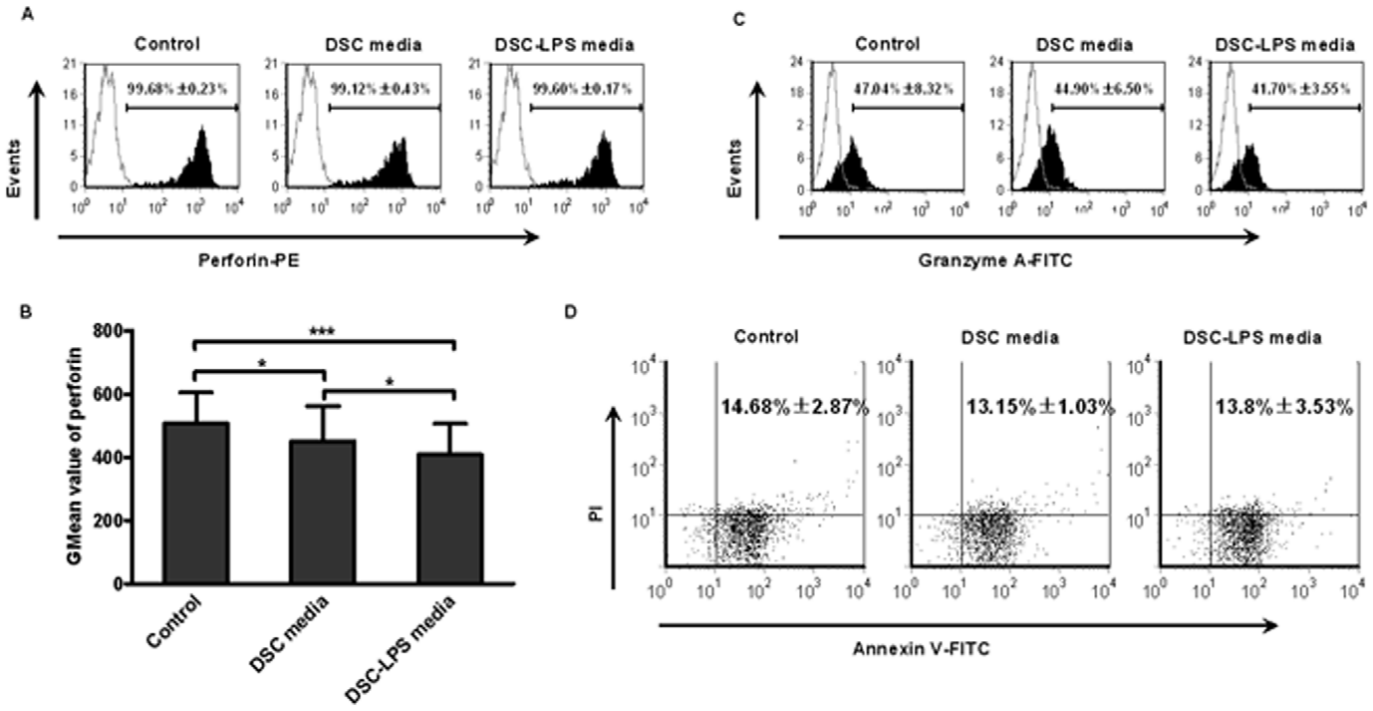
The effects of DSCs media on apoptosis and expression of perforin and GrzA in NK cells. Peripheral CD56^+^ NK cells were incubated with fresh complete media, DSC-conditioned media or DSC-LPS media (prepared as described in *Materials and Methods*) for 48 hours. The apoptosis and intracellular expression of perforin and GrzA were examined by flow cytometry. (A) Flow cytometric analysis of perforin-positive cells. (B) GMean value of perforin expressed in CD56^+^ NK cells exposed to different types of media. (C) The percentage of GrzA-positive cells. (D) Flow cytometric analysis the apoptosis rate of CD56^+^ NK cells. The data shown represent three independent experiments (mean±SD), and the images are from an experiment that was representative of three independently conducted experiments. *P<0.05 and *** *P*<0.0001.

Recently, an increasing focus has been placed on the immunomodulatory function of DSCs due to their close contact with the immune cells. Therefore, we sought to determine what effect, if any, DSCs had on NK cells. CD56^+^ NK cells (2×10^5^ cells/mL) from normal female individuals were co-cultured with the supernatants from DSCs. After 48 hours, the levels of perforin and GrzA expression in CD56^+^ NK cells were measured by flow cytometry. Fig. 2A showed that both groups express perforin at a high level (>98%). However, the GMean value of CD56^+^ NK cells treated with DSC-conditioned media (452.19±110.36) was profoundly attenuated compared with that of the control (508.37±97.18) (Fig. 2B), suggesting that factors in the DSC-conditioned media inhibit perforin expression in CD56^+^ NK cells. The results presented in Fig. 2C indicated that the GrzA expression levels of the two groups were 47.04%±8.32% and 44.9%±6.5%, respectively, and there was no significant difference. To investigate whether TLR4 present on the surface of DSCs regulates the cell-to-cell communication, CD56^+^ NK cells (2×10^5^ cells/mL) were treated with media collected from DSCs that had been stimulated with LPS (1 µg/mL, the dose was determined according to pre-experiment, Figure S1, DSC-LPS media) and then the perforin and GrzA expression were analyzed by flow cytometry.

As shown in Fig. 2B, although there was no difference in the percentages of perforin- and GrzA- positive cells among the groups (Fig. 2A and Fig. 2C), the GMean value of perforin in CD56^+^ NK cells treated with DSC-LPS media (409.28±98.3) was lower than that of cells with DSCs media treatment, suggesting that LPS augmented the inhibitory effect of DSCs on CD56^+^ NK cells. Therefore, the question arose of whether the media inhibited perforin expression directly or by inducing apoptosis of cells. We then examined apoptosis of CD56^+^ NK cells that had been incubated with DSC-conditioned media or DSC-LPS media. The results shown in Fig. 2D indicated that the apoptosis rates of the control, the DSCs media group and the DSC-LPS group were 14.68%±2.87%, 13.15%±1.03% and 13.8%±3.53%, respectively, and there was no significant difference. These data suggested that media from DSCs with or without LPS stimulation directly inhibited perforin expression.

### The effect of LPS on cytokine mRNA expression and protein release of DSCs

**Figure 3:**
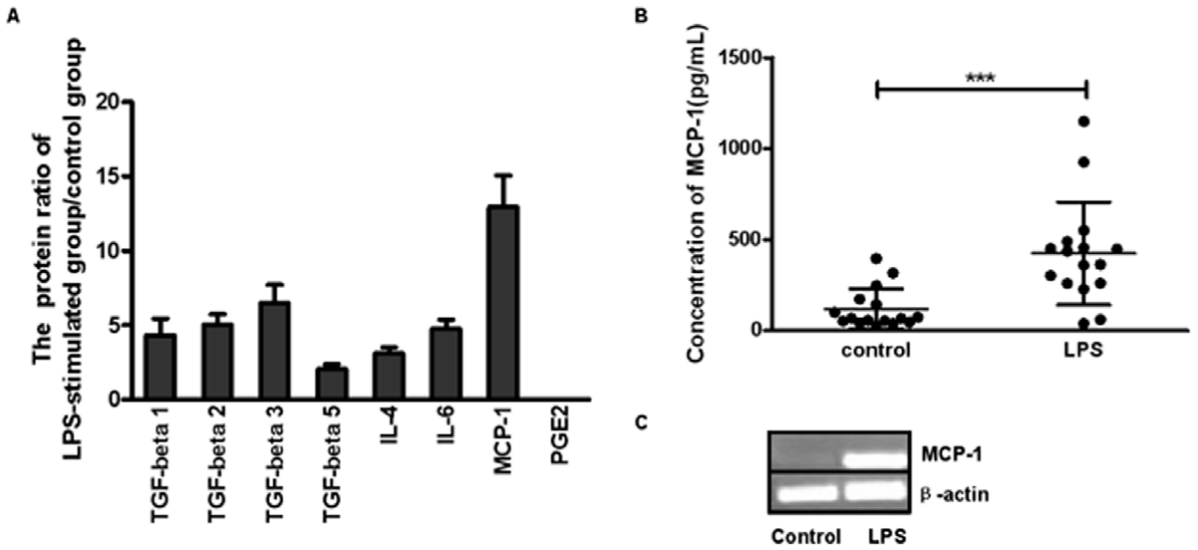
The effect of LPS on cytokine mRNA expression and proteins release from DSCs. (A) DSCs were incubated for 12 hours with or without LPS (1 µg/mL) stimulation. After another 24-hours of culture, the supernatants from the different samples (n = 3) were collected for protein array analysis. The values in the picture represent the standard protein ratio of the LPS-stimulated group to that of control group. (B) The supernatants of DSCs (n = 16) with or without LPS stimulation were harvested, and the protein level of MCP-1 was detected by ELISA. (C) Total RNA from DSCs was extracted, and the MCP-1 mRNA expression was examined by RT-PCR. The picture is from a representative experiment. LPS: DSCs that were preincubated with LPS (1 µg/mL). *** *P*<0.0001.

To identify the key factor that was regulated by LPS and affected the interaction of DSCs and CD56^+^ NK cells, we measured the expression levels of several cytokines that have been reported as inhibitory or modulatory factors of the immune system, including PGE_2_, TGF-β, IL-4, IL-6, MCP-1 [18], [19]. DSCs (5×10^5^ cells/mL) were seeded onto 6-well plates and incubated with or without LPS for 12 hours and then washed. After another 24 hours of culture, the supernatants from 3 samples were harvested for protein array analysis (RayBio® Biotin Label-based Human Antibody Array I). Assays based on cytokine antibody arrays were carried out according to the manufacturer's instructions (KangChen Inc., Shanghai, China). Fig. 3A showed that the TGF-β family, IL-4 and IL-6 were all up-regulated to various extents by LPS. However, the change in the level of MCP-1 secretion was much greater (12.949±2.109 folds). We did not detect a change in PGE_2_ expression using this method. We further confirmed that MCP-1 expression of DSCs by RT-PCR and ELISA. Fig. 3C indicated that the expression of MCP-1 was profoundly augmented by LPS at the transcriptional level, and Fig. 3B showed that although results were heterogeneous, the amount of MCP-1 secreted into the media by DSCs was significantly increased in the presence of LPS.

### MCP-1 present in the media of DSCs with or without LPS stimulation inhibited perforin expression in CD56^+^ NK cells

The substantial changes in MCP-1 expression of DSCs resulting from LPS stimulation suggest that it may be the factor that mediates the inhibitory effect of DSCs on CD56^+^ NK cells and is regulated by LPS. Therefore, we conducted another experiment to test this hypothesis. Freshly isolated CD56^+^ NK cells (2×10^5^ cells/mL) were stimulated as follows: media from DSCs, DSC-LPS media and rMCP-1, for 48 hours. An anti-MCP-1 neutralizing mAb (10 µg/mL) was added to the media 1 hour prior to the stimulation, and normal goat IgG (10 µg/mL) was used as isotypic control.

**Figure 4:**
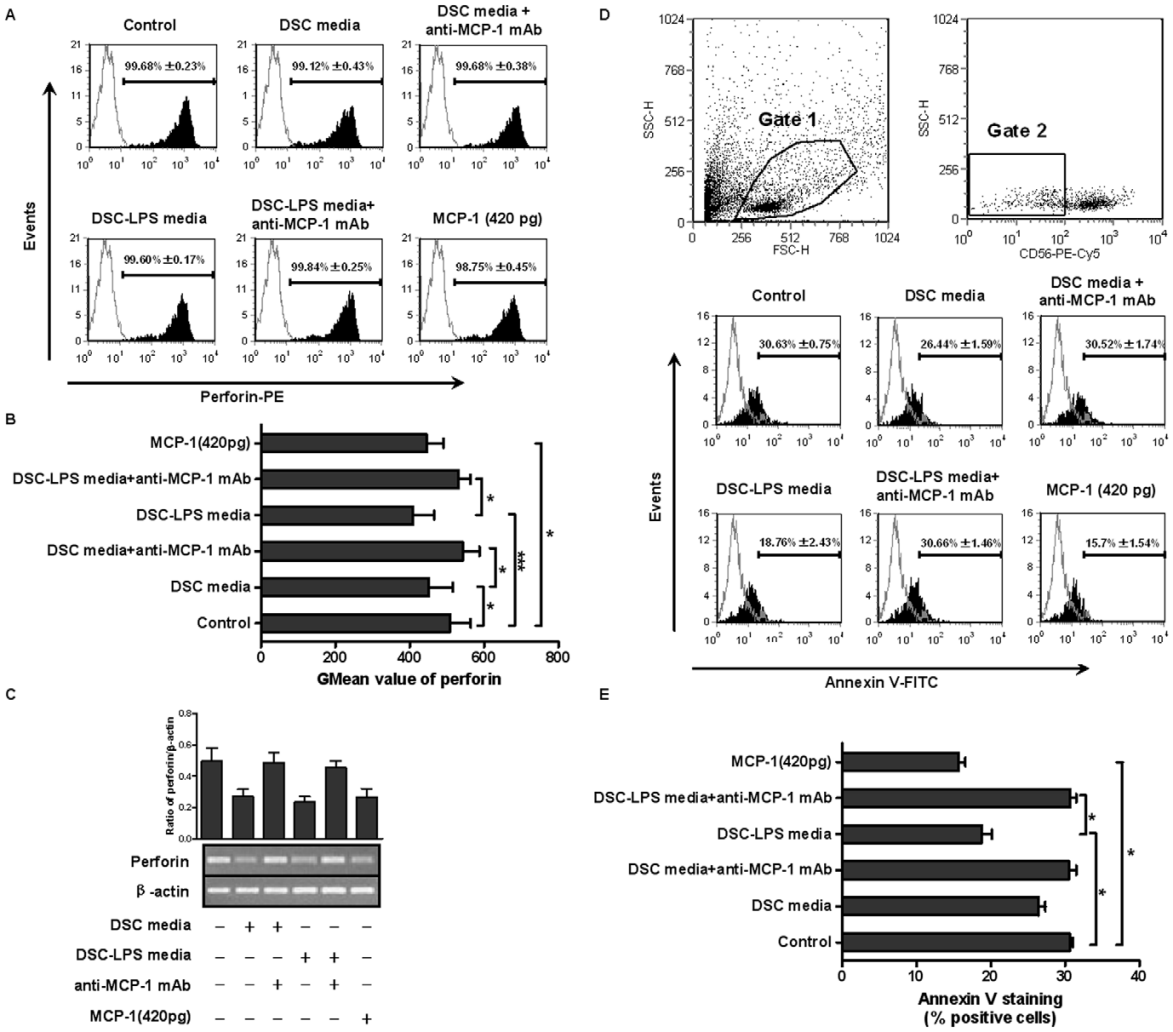
DSC-derived MCP-1 was involved in the inhibition of perforin expression in CD56^+^ NK cells. Freshly isolated CD56^+^ NK cells were stimulated with DSC media, DSC-LPS media or MCP-1 (420 pg/mL, according to the mean level of 16 DSC-LPS media detected by ELISA) for 48 hours. An anti-MCP-1 neutralizing mAb was added to the cultures 1 hour prior to the stimulation, and the perforin expression was analyzed by flow cytometry and RT-PCR. (A) Flow cytometric analysis of perforin. The picture is from a representative experiment, and the numbers are the percentages of positive cells. (B) GMean value of perforin expression. (C) A representative graph of perforin mRNA expression detected by RT-PCR. Respective density analysis of the bands normalized to β-actin bands. (D) Flow cytometric analysis of apoptosis of K562 cells induced by NK cells. NK cells indifferent treatment conditions were incubated with K562 (NK∶ K562 ratio: 1∶4) for 5 hours. The cytotoxicity of he NK cells was evaluated based on the percentage of CD56-negative (K562 cells) annexin V-positive cells as previously described. Gate 1, cells involved in our analysis. Gate 2, CD56-negative cells. (E) Graphs represented percentage of annexin V-positive cells. All data shown were expressed as mean±SD of 3 independent experiments. *P<0.05 and *** *P*<0.0001.

As previously described, there was no difference in the percentage of perforin- positive cells (>98%)( Fig. 4A). Media from DSCs induced a decrease in the GMean value for perforin expression in CD56^+^ NK cells, and DSC-LPS media resulted in a further decrease. However, Fig. 4B showed that anti-MCP-1 neutralizing mAb could restore the GMean value for perforin expression in CD56^+^ NK cells incubated with DSC media (544.1±75.98) or DSC-LPS media (531.57±55.41), and the values were even higher than that of the control group (although no significant difference was detected), suggesting that MCP-1 secreted by DSCs attenuated perforin expression in CD56^+^ NK cells. Normal goat IgG had no effect on perforin expression. To further confirm the effect of MCP-1, rMCP-1 was added in the cultures, and we found that rMCP-1 profoundly decreased the GMean value of perforin expression (445.45±78.39). Fig. 4C indicated that DSC-conditioned media, DSC-LPS media and rMCP-1 inhibited mRNA expression of perforin in CD56^+^ NK cells, and the addition of an anti-MCP-1 mAb abolished the effects of both media. Together, these results suggested that MCP-1 suppressed expression of perforin in NK cells at both the protein and mRNA level.

To further investigate whether the reduced expression of perforin may influence the cytotoxicity of NK cells, we performed co-culture experiments with CD56^+^ NK cells and K562 cells (NK∶ K562 ratio: 1∶4) and examined the cytolytic potential of NK cells with different stimulation. As shown in Fig. 4D and Fig. 4E, the apoptosis of K562 cells (CD56-negative and annexin V-positive cells) induced by NK cells in control, DSC-LPS media and rMCP-1 groups were 30.63%±0.75%, 18.76%±2.43%, 15.7%±1.54%, respectively. The administration of anti-MCP-1 antibody reversed the apoptosis rate in the DSC-LPS media group. Interestingly, although treatment with DSC media resulted in a subtle decrease in apoptosis in K562 cells (26.44%±1.59%) compared with the control (30.63%±0.75%), there was no significant difference between the two groups.

### MCP-1 inhibited perforin production in CD56^+^ NK cells by up-regulating SOCS3 expression

**Figure 5:**
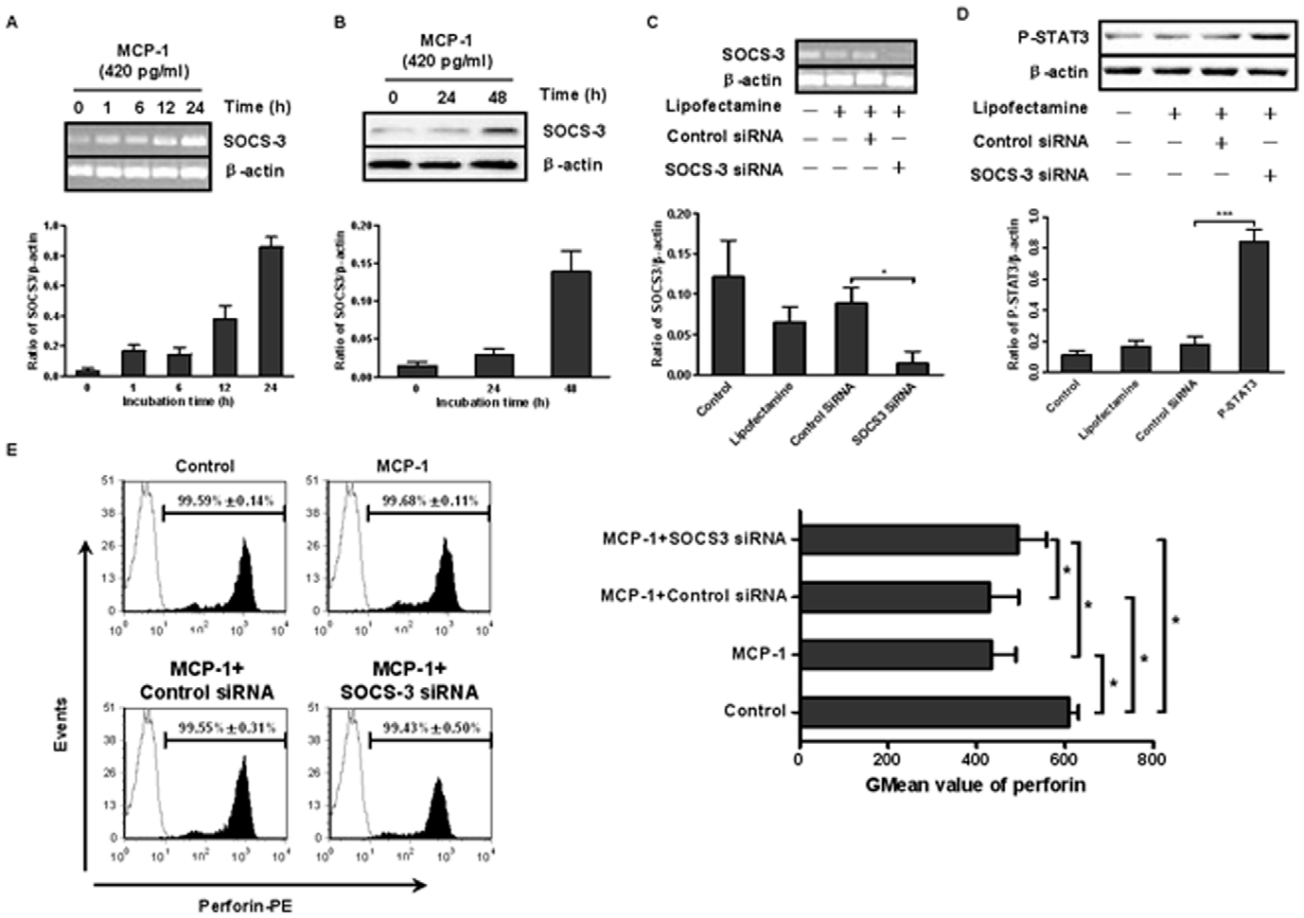
MCP-1 inhibited perforin production in NK cells by up-regulating SOCS3 expression. Isolated CD56^+^ NK cells were transfected with control siRNA or SOCS3 siRNA (at a final concentration of 1 µM) for 48 hours. (A) NK cells with SOCS3 silencing were treated with rMCP-1 (420 pg/mL). The mRNA expression of SOCS3 at different points was detected by RT-PCR, and the respective density was analyzed. (B) The effect of MCP-1 on protein expression of SOCS3 at 0 hours, 24 hours and 48 hours was detected by western blot, and the respective density was analyzed. (C) and (D) 48 hours after siRNA transfection, the efficacy of SOCS3 siRNA was evaluated. The expression of SOCS3 mRNA was detected by RT-PCR, and the expression of SOCS3 protein was indirectly examined by phospho-STAT3 detection. Increased phospho-STAT3 levels represented a reduction of SOCS3 expression. An analysis of the respective densities of the bands normalized to β-actin bands was conducted. (E) Transfected NK cells were stimulated with MCP-1 (420 pg/mL) for 48 hours, and the perforin expression was analyzed by flow cytometry. The percentages of perforin-positive cells and their corresponding GMean values are presented in the figure. All of the graphs are representive, and all data shown are expressed as the mean±SD of 3 independent experiments. *P<0.05.

It has been reported that cytokines can induce SOCS3 expression [20], and overexpression of SOCS3 in mice was shown to reduce the level of perforin in the liver [21]. Therefore, we sought to investigate whether SOCS3 was involved in the inhibition of perforin that was induced by MCP-1. CD56^+^ NK cells were treated with rMCP-1 (420 pg/mL) for different lengths of time, and the expression of SOCS3 was detected by RT-PCR and western blot. As shown in Fig. 5A, SOCS3 mRNA expression was up-regulated as early as 1 hour after the addition of MCP-1, and the level continued to increase at the later time points. A low level of SOCS3 protein was detected. The basic expression of SOCS3 protein in CD56^+^ NK cells was detected at a low level. However, the expression was induced at 24 hours after MCP-1 stimulation, peaking at 48 hours (Fig. 5B). Together, these results suggested that SOCS3 expression was influenced by MCP-1, and it was likely involved in the regulation of perforin expression.

To further investigate the role of SOCS3, we treated CD56^+^ NK cells with SOCS3 siRNA. Fig. 5C showed that SOCS3 siRNA induced a prominent decrease at the level of SOCS3 mRNA expression, and lipofectamine alone and a control siRNA had no effect on SOCS3 mRNA levels. Fig. 5D indicated that cells with SOCS3 silencing showed a stronger phospho-STAT3 signal, which represented a reduced expression of SOCS3. These results confirmed the efficacy of SOCS3 siRNA. Next, we examined the effect of MCP-1 on NK cells with SOCS3 silencing and found that compared with the control siRNA group (430.59±65.39), the GMean value of perforin expression in NK cells pretreated with SOCS3 siRNA was higher (493.31±65.02) after MCP-1 stimulation. However, the value was still lower than that of the control (609.45±21.10), which suggests that MCP-1 may exert inhibitory effect partly through SOCS3.

### Both the NF-κB and ERK/MAPK pathways were involved in LPS-stimulated MCP-1 up-regulation in DSCs

The effect of MCP-1 on the interaction between DSCs and CD56^+^ NK cells led us to investigate the regulatory mechanism of LPS-stimulated MCP-1 production. We preincubated DSCs with inhibitors of several signaling molecules for 1 hour before LPS stimulation. After 24 hours, the supernatants were collected for ELISA. Fig. 5A showed that MCP-1 production induced by LPS was dramatically impaired by pretreatment with PDTC (4 µg/mL) and U0126 (10 µM). However, wortmannin (100 nM) did not influence the MCP-1 expression in cells stimulated with LPS.

**Figure 6:**
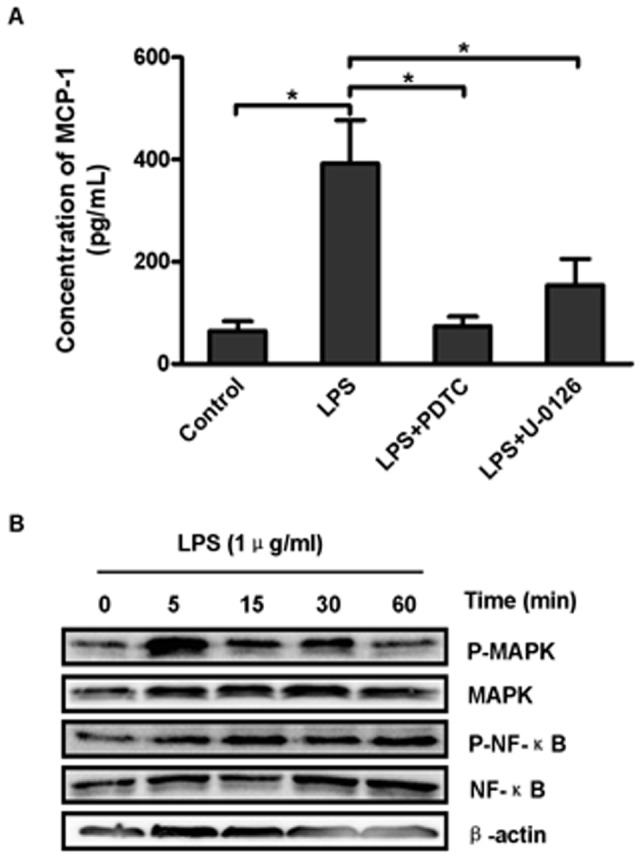
The NF-κB and ERK/MAPK pathway were both involved in the LPS-stimulated up-regulation in DSCs. (A) DSCs were pretreated with PDTC (4 ug/mL), U-0126 (10 µM) or wortmannin (100 nM) for 1 hour and then cultured with LPS (1 µg/mL) for 24 hours. MCP-1 in the supernatant was detected by ELISA. The results shown are the mean±SD of 3 independent experiments. *P<0.05 and *** *P*<0.0001. (B) DSCs were stimulated with LPS (1 µg/mL), and the cells were harvested at 0 minutes, 5 minutes, 15 minutes, 30 minutes and 60 minutes for western blot analysis. The image shown is representative of 3 independent experiments. (C) The graphs represent the quantitation of different signaling molecules using β-actin as an internal standard. The data are shown as the mean ± SD (n = 3).

We then examined the signaling molecules involved by western blot analysis, and the kinetics of NF-κB and ERK activation were investigated. Total proteins were extracted at 0 minutes, 5 minutes, 15 minutes, 30 minutes and 60 minutes after LPS stimulation. As shown in Fig. 6B and Fig. 6C, during LPS stimulation, MAPKp44/42 activation reached a peak value as early as 5 minutes, and it presented a much lower level from 15 minutes to 30 minutes. By 60 minutes of LPS stimulation, the level of phospho-MAPKp44/42 had declined to the level detected at 0 minutes. In contrast, NF-κB phosphorylation became evident as early as 5 minutes, rose moderately at 15 minutes, and remained steady until 60 minutes. Taken together, these findings suggested that both NF-κB and ERK activation are associated with up-regulation of MCP-1in DSCs stimulated by LPS.

## Discussion

During a successful pregnancy, a unique structural organization forms called the decidua, which allows an embryo expressing paternal antigens to implant and cohabitate with the mother without being rejected by the maternal immune system. Leukocytes seem to play a role in feto-maternal immune adjustment and DSCs are important for the education of leukocytes [7]. However, the factors that mediate this cell-to-cell communication are not fully understood. In the present study, we explored the effect of DSCs on CD56^+^ NK cells, which are closely related with pregnancy, and we further investigated the related modulatory mechanisms.

It has been reported that DSCs play important roles in feto-maternal tolerance during a successful pregnancy [5]. A recent report indicates that DSCs are endowed with the capacity to regulate cytokines production of T cells as non-professional APCs [7]. Other reports show that DSCs are able to produce several cytokines that recruit immune effector cells and regulate their differentiation and activation [22], [23]. Our study reveals that media from DSCs suppressed perforin expression in CD56^+^ NK cells without affecting GrzA expression. Although we detected GrzB expression during our studies, the results were inconclusive (data not shown), and the effect of DSC-conditioned media on GrzB expression needs to be further studied. Perforin is a type of cytotoxic granule found in the cytoplasm of NK cells. When challengers are encountered, NK cells exocytose perforin and granzyme to lyse the target cells [24], [25]. Thus, the inhibitory effect exerted by DSCs media on perforin production in NK cells could cause NK cells to exhibit a lower level of cytotoxicity. As expected, DSC-LPS media, as well as rMCP-1, significantly inhibited apoptosis in K562 cells induced by NK cells. Interestingly, DSC-conditioned media failed to produce a statistically significant suppression of the cytotoxic activity of NK cells. One possibility is that the inhibitory effect of DSC-conditioned media on perforin expression is not strong enough to affect the cytotoxicity of NK cells. Our results are consistent with a previous study which demonstrated that TGF-β released by DSCs promotes the conversion of peripheral blood NK cells into less cytotoxic uterine NK cells [8]. The regulation of peripheral NK cells by DSCs may be a source of uterine NK cells that play a vital role in the invasion, proliferation and differentiation of the trophoblast [2]. Additionally, both DSCs media did not influence the apoptosis of cultured CD56^+^ NK cells, suggesting that the inhibition of perforin expression is a direct effect and is not due to an increased apoptosis.

In the present study, we confirmed the surface expression of TLR4 by flow cytometry and demonstrated its transcription by RT-PCR in first-trimester DSCs after 3–4 passages. TLR4 is the specific receptor for gram-negative bacterial LPS. Traditionally, TLRs are considered to be correlated with infection. However, recently an increasing focus has been placed on immunomodulation of decidual TLR4 recently. Factors released from trophoblast cells in response to TLR4 activation are able to enhance recruitment of immune cells and modulate their function [26], [27], and dysfunction of TLR4 is associated with several pregnancy complications such as pre-eclampsia and eclampsia [28], [29], [30], [31]. Our results also show that DSC-LPS media has a stronger inhibitory effect on perforin expression in CD56^+^ NK cells. All of these results indicate that TLR4 plays a role in the suppression of DSCs during successful pregnancy.

MCP-1, the first chemokine with a C-C motif to be identified, is able to recruit monocytes/macrophages, T cells, basophiles and NK cells to inflammatory sites [32], [33]. Moreover, MCP-1 works as a double-edged sword to regulate immune response. Previous research demonstrates that MCP-1 could promote the development of polarized Th2 responses [18], [34] and participate in diseases related to Th2 status [35], which underlines the significance of MCP-1 in the Th2 response. Interestingly, Th2 polarization is required for successful pregnancy at the feto-maternal interface, and MCP-1 is highly expressed in the decidua during early pregnancy [36]. In this study, we report for the first time that compared with several other modulatory cytokines, MCP-1 expression in DSCs is profoundly up-regulated by LPS pretreatment, suggesting that MCP-1 may play a role in the crosstalk between DSCs and CD56^+^ NK cells. As expected, an anti-MCP-1 neutralizing antibody can restore the inhibitory effect exerted by media from DSCs on perforin expression in CD56^+^ NK cells, and rMCP-1 similarly suppresses perforin expression just as DSCs media. It has been reported that MCP-1 induces migration in IL-2 (250 U/mL) long-term activated NK cells, whereas it only shows a weak influence on resting NK cells [37]. IL-2 is an effective activator of NK cells. In addition to maintaining the growth and proliferation of NK cells, long-term stimulation with IL-2 may result in enhanced cytotoxicity of NK cells. The selective chemotaxis of MCP-1 on NK cells may contribute to their suppression and maintain a local immune balance. However, a previous report shows that MCP-1 augments the cytotoxicity of NK cells against PC-14 tumor cells and suppresses tumor spread in mice [38]. The discrepancy might be related to species variation, the global immune condition of the research subjects as well as to differences in the experimental methods used and the molecules detected. Previous research has indicated that cytokines could induce SOCS3 expression [20]. Consistently, our results suggest that SOCS3 expression is up-regulated by MCP-1 at both the mRNA and protein levels. We also found that silencing of SOCS3 partly prevented MCP-1 from suppressing perforin expression in NK cells. This finding is in line with a previous report indicating that forced expression of SOCS3 in mice T cells leads to a decrease of perforin in the liver [21]. SOCS3 is a negative feedback inhibitor of STATs family. Interestingly, the perforin expression is closely related to STAT3 and STAT5, etc [39], [40]. The overexpression of SOCS3 induced by MCP-1 may lead to suppression of perforin in NK cells by inhibiting activity of STAT family members. However, there must be other molecules involved in the suppression of perforin caused by MCP-1, as the silencing of SOCS3 only partly abolishes the effect of MCP-1, and further study is needed to explore the potential alternative pathway.

LPS stimulates MCP-1 and RANTES production in mouse primary renal tubular epithelial cells [41], and stimulation of human uterine epithelial cell with LPS results in up-regulation of IL-8 and MCP-1 [42]. Upon ligand binding, the TLRs recruit intracellular adaptor molecules such as MyD88, IRAK and TRAF6, and the recruitment of these molecules results in the activation of a subsequent kinase cascade, including the phosphorylation of MAPKs and the activation of the NF-κB pathway, and the resultant production of cytokines or other inflammatory mediators [15], [43]. In the present study, we demonstrate for the first time that LPS-TLR4 signals through both NF-κB and the ERK/MAPK pathway in first-trimester DSCs, and this part of the signaling pathway may play an important role in regulation of the local immune system because DSCs are directly exposed to gram-negative bacteria in the reproductive tract. It must be noted that LPS treatment induces significant decidual stromal cell death at a concentration of 1 µg/mL, as shown in a previous work [44]. However, we did not observe any significant difference in the cell morphology or viability between the control group and the LPS-treatment group (data not shown).

**Figure 7:**
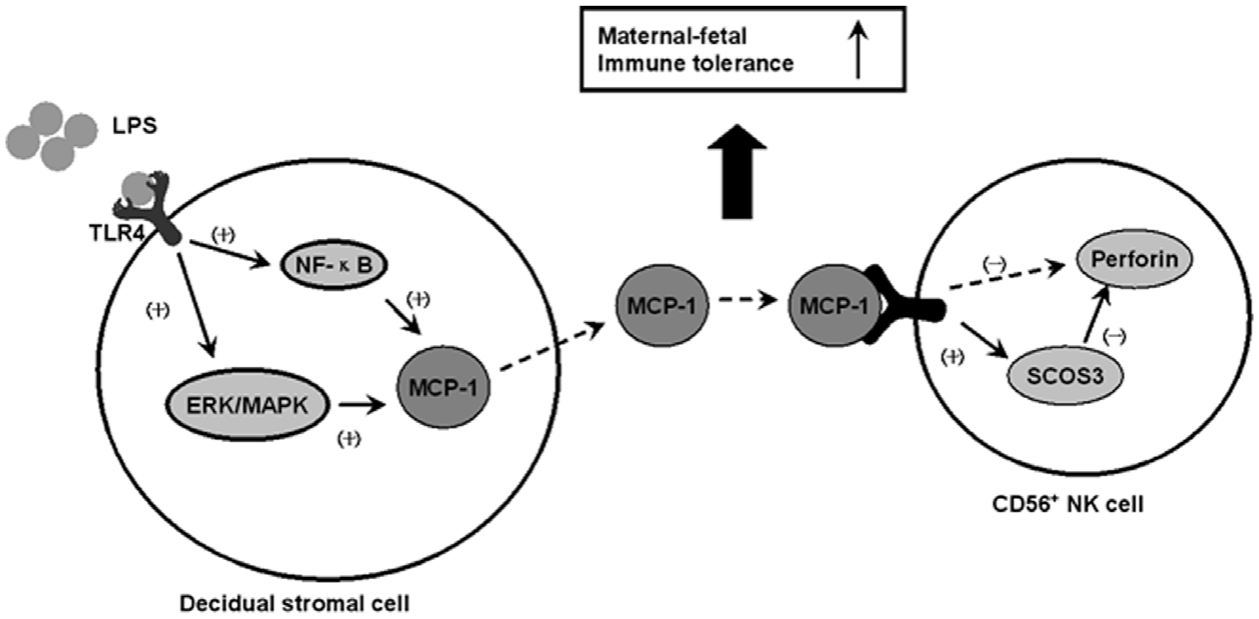
Modulatory model representing the effect of DSCs on CD56^+^ NK cells, which is mediated by MCP-1. Gram-negative bacteria that parasitize the reproductive tract can be recognized by TLR4 expressed on DSCs, and the ligation of bacterial LPS with TLR4 leads to activation of the ERK/MAPK and NF-κB pathways, which in turn results in up-regulation of MCP-1 in DSCs. Secreted MCP-1 inhibits perforin production in NK cells by promoting SOCS3 expression, which may contribute to the fetal-maternal tolerance.

Our results support the possibility of a modulatory mechanism of immune cells during early pregnancy. As shown in Fig. 7, gram-negative bacteria parasitizing reproductive tract during a normal pregnancy can be recognized by TLR4 expressed on DSCs of the first-trimester deciduas. The ligation of bacterial LPS with TLR4 leads to activation of the ERK/MAPK and NF-κB pathways, which in turn stimulate the generation of MCP-1. MCP-1 secreted from DSCs up-regulates SOCS3 expression in CD56^+^ NK cells, which consequently inhibits perforin expression and results in a decrease of NK cells cytotoxicity. These actions together were to promote the feto-maternal tolerance. This model places the DSCs at the center of the immunomodulation mechanism and elucidates the significance of MCP-1, which is highly expressed at the feto-maternal interface during early pregnancy. However, the regulatory effect of DSCs on other immune cells needs to be further investigated. Notably, most previous studies have focused on the relationship between pathological pregnancy, such as pre-eclampsia or eclampsia with intrauterine growth restriction, and TLRs. Actually, the level of LPS and TLR4 are usually abnormally elevated under pathological conditions. In the current study, we explore the expression of TLR4 under physiological condition as well as the corresponding mechanism involved in the defense infection and establishment of tolerance, as hypothesize by Gil Mor and co-worker [43]. Our data may provide a novel clue for therapeutic approaches during pathological pregnancies.

In summary, we have demonstrated that during the first trimester, DSCs are able to suppress perforin expression in CD56^+^ NK cells. This effect is mediated by MCP-1 and enhanced by LPS. MCP-1 exerts an inhibitory effect by up-regulating SOCS3 expression in NK cells, and LPS could stimulate MCP-1 production in DSCs through both the ERK/MAPK and NF-κB pathways. The crosstalk between DSCs and CD56^+^ NK cells mediated by MCP-1 may be a crucial modulatory mechanism of the maternal immune system to maintain feto-maternal tolerance during pregnancy.

## Materials and Methods

### mAbs and reagents

The mouse anti-human vimentin monoclonal antibody (mAb) used for immunocytochemistry was obtained from Dingguo (Beijing, China), and anti-cytokeratin-7 was obtained from Zymed Laboratories (San Francisco, CA). The fluorescein isothiocyanate (FITC)-conjugated mouse anti-human GrzA mAb (CB9) was purchased from Biolegend (San Diego, CA, USA). The phycoerythrin (PE)-conjugated mouse anti-human perforin (dG9) mAb, the allophycocyanin (APC)-conjugated mouse anti-human TLR-4 mAb and the MCP-1 commercial ELISA kit were obtained from eBioscience (Hatfield, UK). The rabbit anti-human NF-κB, anti-human phospho-NF-κB, anti-human MAPKp44/42, anti-human phospho-MAPKp44/42, anti-human SOCS3, anti-human phosphor-STAT3 antibodies, the U0126 (inhibitor of ERK1/2) and wortmannin (inhibitor of PI3K) were obtained from Cell Signaling Technology (Boston, MA, USA). The mouse anti-human β-actin mAb was obtained from Jingmei (Shenzhen, China). The goat anti-human MCP-1 neutralizing antibody and the recombinant human MCP-1 (rMCP-1) were obtained from R&D Systems (Minneapolis, MN). All isotype controls were purchased from the corresponding vendors. LPS from *Escherichia coli*. (055.B5) was purchased from Sigma (St. Louis, MO, USA). Pyrrolidine dithiocarbamate (PDTC; inhibitor of NF-κB) was obtained from Calbiochem (La Jolla, CA, USA).

### Isolation and culture of human first trimester DSCs

Decidual samples were obtained from 16 healthy women undergoing elective vaginal termination of their pregnancies (6–8 weeks) at Qilu Hospital, Shandong University. Peripheral blood was collected from normal young female donors. Our study was approved by the Ethics Committee of Qilu Hospital, Shandong University. Written informed consent was obtained from all subjects.

First trimester decidual stromal cells were purified and cultured using a protocol that has been previously described [5]. Briefly, fresh decidual tissues collected in sterile medium were rinsed in cold sterile RPMI 1640 (Invitrogen, Carlsbad, CA) medium 3 times and then minced into small pieces. The pieces were digested with 0.1% collagenase type I (Sigma) and 0.25% trypsin (Invitrogen) for 20 minutes in a 37°C shaking water bath, and the process was terminated by the addition of RPMI 1640 medium containing 10% new-born calf serum. The digestion was subjected to filtration through a sieve (75 and 125 µm), and the samples were centrifuged at 120×g for 10 minutes. The supernatant was discarded, and the cell pellet was resuspended and seeded in culture flasks in complete RPMI 1640 medium containing 10% fetal bovine serum (GIBCO). After 1 hour, the macrophages and granulocytes adhereing to the flasks were discarded and the supernatant was cultured overnight. Then, the medium was replaced, and the blood cells in the supernatant were discarded. After 3–4 passages for approximately 1–2 weeks with the culture medium being replaced every 48 hours, decidual cells isolated during the first trimester were confirmed to be vimentin-positive (>98%) and cytokeratin-negative by immunocytochemistry, which is consistent with published results [3].

DSCs (5×10^5^/mL) were incubated in complete RPMI 1640 medium in the presence of LPS (1 µg/mL) for 12 hours and then washed to remove the LPS. After another 24 hours of culture, the supernatant was centrifuged at 780×g for 10 minutes and immediately stored at −80°C for future use. Meanwhile, the cells were dissociated with Trizol (Shenggong,Shanghai, China) for RT-PCR. In some cases, PDTC (4 µg/mL), U0126 (10 µM) or wortmannin (100 nM) were added to confluent DSCs 1 hour prior to LPS treatment.

### NK cell isolation, culture, transfection and treatment

Peripheral blood mononuclear cells were isolated by Ficoll density gradient centrifugation (Sigma-Aldrich, St. Louis, MO, USA). From these samples, CD56^+^ NK cells were isolated by positive selection with anti-CD56 microbeads (MACS, Miltenyi Biotec) following the manufacturer's instructions. These cells were immediately cultured in complete RPMI 1640 medium containing 10% fetal bovine serum plus 100 U/mL rIL-2. To silence SOCS3, a small interfering RNA (siRNA) targeting human SOCS3 designed as previously reported [45] (sense : 5-CCAAGAACCUGCGCAUC-CAdTdT-3, antisense: 5-UGGAUGCGCAGGUUCUUGGdTdT-3) was purchased from Invitrogen and a nontargeting siRNA was used as a control (Dharmacon). The NK cells were transfected with siRNA (at a final concentration of 1 µM) for 48 hours using Lipofectamine 2000 (Invitrogen) reagent. NK cells with or without SOCS3 silencing were then treated as follows: DSC-conditioned media, DSC-LPS media, rMCP-1 (420 pg/mL, according to mean value of 16 DSC-LPS media detected by ELISA). In some cases, the cells needed to be preincubated for 1 hour with an anti-MCP-1 neutralizing antibody (10 µg/mL) before the treatment. After 48 hours of culture, the supernatants were harvested, centrifuged at 780×g for 10 minutes and then stored at −80°C. The cells were used for flow cytometric analysis.

### Evaluation of NK cells cytotoxicity

To detect the effect of MCP-1 as well as that of the different DSC media on the cytotoxic activity of NK cells, CD56^+^ cells were isolated and treated with DSC media, DSC-LPS media, or MCP-1 (420 pg/mL) in the absence or presence of an anti-MCP-1 antibody and then cultured with K562 target cells (kindly provided by Department of Hematology and Oncology, Qilu Hospital of Shandong University [46]) for 5 hours (NK∶ K562 ratio: 1∶4). Then, the cells were collected, stained with an anti-CD56 mAb and Annexin V and analyzed by flow cytometry. The cytolytic potential of the NK cells was evaluated based on the percentage of CD56^−^ (K562 cells) Annexin V-positive cells as previously described [47].

### Western blot analysis

The LPS signaling pathway in DSCs, as well as efficacy of SOCS3 siRNA in NK cells was detected by western blot. However, the SOCS3 western blot signal in resting NK cells was very low, so we used phospho-STAT3 as an indicator of SOCS3 silencing efficacy according to a previous report [48]. Cultured cells were harvested and lysed immediately. Total protein (40 µg) was separated with 10% sodium dodecyl sulfate polyacrylamide gels and then transferred onto nitrocellulose membranes. Rabbit anti-human NF-κB (1∶1000), anti-human phospho-NF-κB (1:1000), anti-human MAPKp44/42 (1∶2000), anti-human phospho-MAPKp44/42 (1∶2000), anti-human SOCS3 (1∶1000) and anti-human phospho-STAT3 (1∶1000) antibodies were used to probe their substrates overnight at 4°C, and a specific horseradish peroxidase-conjugated goat anti-rabbit secondary antibody was used to blot the target proteins. The immunoreactivity of target proteins was detected by an enhanced chemiluminescence ECL detection kit as described.

### Immunocytochemistry

We determined the purity of the DSCs by immunocytochemistry. After 3–4 passages, the DSCs were seeded on coverslips, fixed in acetone and methanol (1∶1 fixed) for 15 minutes at 4°C and washed in PBS. Then, 0.3% H_2_O_2_ and 0.1% normal rabbit serum in PBS were used to block endogenous peroxidase and non-specific binding of antibodies, respectively. The cells were incubated overnight at 4°C in the presence of a mouse anti-human vimentin monoclonal antibody or a cytokeratin antibody (1∶100 diluted). Normal mouse IgG was used as an isotypic control. After rinsing three times with PBS, the slides were incubated with a biotin-conjugated secondary antibody for 15 minutes at 37°C and washed three times. The slides were then incubated with avidin/peroxidase solutions, stained with DAB and counterstained with Mayer's hematoxylin (Sigma).

### RT-PCR

Total RNA was extracted from the cultured cells with Trizol reagent. One microgram of total RNA was reverse-transcribed in a 20 µl volume using an RT-PCR kit (Fermentas Life Science, USA), according to the manufacturer's instructions. Reverse transcription-polymerase chain reaction (RT-PCR) was performed as described previously. Amplified samples (10 µL) were electrophoresed on 1% agarose gels containing ethidium bromide and photographed. The primer sequences are as follow: MCP-1: sense 5′-CTCAGCCAG-ATGCAATCAATGC-3′, antisense 5′-CCTCAAGTCTTCGGAGTTTGGG-3′; TLR4: sense 5′ –GTGGAAGTTGAACGAATGGA-3′, antisense 5′-TGGATGATGTTGGCAGCA-3′; perforin: sense 5′-AAAGTCAGCTCCACTGAAGCTGTG-3′, antisense 5′-AGTCCTCCACCTCGTTGTCCGTGA-3′; SOCS3: sense 5′ –GGACCAGCGCCACTTCTTCAC-3′, antisense 5′-TACTGGTCCAGGAACTCCCGA-3′; β-actin: sense 5′-AGCGAGCATCCCCCAAAGTT-3′, antisense 5′- GGGCACGAAGGCTCATCATT-3′.

### ELISA

Supernatants of the DSCs prepared using the method described above were used to determine the response of the DSCs to LPS. The level of MCP-1 was detected using a commercial ELISA kit according to the manufacturer's instruction.

### Flow cytometric analysis

We used a cell fixation/permeabilization kit (eBioscience) to detect the intracellular expression of perforin and GrzA in CD56^+^ NK cells. After 20-minutes of fixation in the dark, CD56^+^ NK cells prepared as described above were incubated with a PE-conjugated anti-perforin mAb (20 µL/10^6^ cells) and a FITC-conjugated anti-GrzA mAb (20 µL/10^6^ cells) for 40 minutes at room temperature. Then, the cells were resuspended in the permeabilization buffer and analyzed by FACSCalibur (BD Biosciences). The surface expression of TLR4 on the DSCs was also detected by flow cytometry.

### Statistical analysis

Data from independent experiments were presented as the mean±SD. S paired t-test or repeated-measures ANOVA were used for statistical evaluations. *P*<0.05 was considered as statistically significant.

## Supporting Information

Figure S1 **LPS stimulates MCP-1 mRNA expression in DSCs in a dose-dependent fashion.** DSCs cells (5×105 cells/mL) were seeded onto 6-well plates, and incubated with LPS of different concentration (10 ng/mL, 100 ng/mL, 1 µg/mL, 10 µg/mL) for 24 hours. The mRNA expression was detected by RT-PCR. The image shown is representative of 3 independent experiments and the graph represents the quantitation of the bands using β-actin as an internal standard. The data are shown as the mean ± SD (n = 3).
(TIF)

## References

[1] Dudley DJ (1997) Pre-term labor: an intra-uterine inflammatory response syndrome? J Reprod Immunol 36: 93–109. 9430741 10.1016/s0165-0378(97)00065-x

[2] Saito S, Shiozaki A, Sasaki Y, Nakashima A, Shima T, et al (2007) Regulatory T cells and regulatory natural killer (NK) cells play important roles in feto-maternal tolerance. Semin Immunopathol 29: 115–122. 17621697 10.1007/s00281-007-0067-2

[3] Kayisli UA, Selam B, Guzeloglu-Kayisli O, Demir R, Arici A (2003) Human chorionic gonadotropin contributes to maternal immunotolerance and endometrial apoptosis by regulating Fas-Fas ligand system. J Immunol 171: 2305–2313. 12928375 10.4049/jimmunol.171.5.2305

[4] Riddick DH, Kusmik WF (1977) Decidua: a possible source of amniotic fluid prolactin. Am J Obstet Gynecol 127: 187–190. 831500 10.1016/s0002-9378(16)33248-3

[5] Olivares EG, Montes MJ, Oliver C, Galindo JA, Ruiz C (1997) Cultured human decidual stromal cells express B7-1 (CD80) and B7-2 (CD86) and stimulate allogeneic T cells. Biol Reprod 57: 609–615. 9282998 10.1095/biolreprod57.3.609

[6] Ruiz C, Montes MJ, Abadia-Molina AC, Olivares EG (1997) Phagocytosis by fresh and cultured human decidual stromal cells: opposite effects of interleukin-1 alpha and progesterone. J Reprod Immunol 33: 15–26. 9185073 10.1016/s0165-0378(96)01009-1

[7] Nagamatsu T, Schust DJ, Sugimoto J, Barrier BF (2009) Human decidual stromal cells suppress cytokine secretion by allogenic CD4+ T cells via PD-1 ligand interactions. Hum Reprod 24: 3160–3171. 19729380 10.1093/humrep/dep308

[8] Keskin DB, Allan DS, Rybalov B, Andzelm MM, Stern JN, et al (2007) TGFbeta promotes conversion of CD16+ peripheral blood NK cells into CD16- NK cells with similarities to decidual NK cells. Proc Natl Acad Sci U S A 104: 3378–3383. 17360654 10.1073/pnas.0611098104 PMC1805591

[9] Dudley DJ, Trautman MS, Mitchell MD (1993) Inflammatory mediators regulate interleukin-8 production by cultured gestational tissues: evidence for a cytokine network at the chorio-decidual interface. J Clin Endocrinol Metab 76: 404–410. 8432783 10.1210/jcem.76.2.8432783

[10] Dosiou C, Giudice LC (2005) Natural killer cells in pregnancy and recurrent pregnancy loss: endocrine and immunologic perspectives. Endocr Rev 26: 44–62. 15689572 10.1210/er.2003-0021

[11] Higuma-Myojo S, Sasaki Y, Miyazaki S, Sakai M, Siozaki A, et al (2005) Cytokine profile of natural killer cells in early human pregnancy. Am J Reprod Immunol 54: 21–29. 15948769 10.1111/j.1600-0897.2005.00279.x

[12] Blanco O, Leno-Duran E, Morales JC, Olivares EG, Ruiz-Ruiz C (2009) Human decidual stromal cells protect lymphocytes from apoptosis. Placenta 30: 677–685. 19560201 10.1016/j.placenta.2009.05.011

[13] Huang SJ, Schatz F, Masch R, Rahman M, Buchwalder L, et al (2006) Regulation of chemokine production in response to pro-inflammatory cytokines in first trimester decidual cells. J Reprod Immunol 72: 60–73. 16806486 10.1016/j.jri.2006.03.002

[14] Lockwood CJ, Matta P, Krikun G, Koopman LA, Masch R, et al (2006) Regulation of monocyte chemoattractant protein-1 expression by tumor necrosis factor-alpha and interleukin-1beta in first trimester human decidual cells: implications for preeclampsia. Am J Pathol 168: 445–452. 16436659 10.2353/ajpath.2006.050082 PMC1606506

[15] Akira S, Takeda K (2004) Toll-like receptor signalling. Nat Rev Immunol 4: 499–511. 15229469 10.1038/nri1391

[16] Krikun G, Lockwood CJ, Abrahams VM, Mor G, Paidas M, et al (2007) Expression of Toll-like receptors in the human decidua. Histol Histopathol 22: 847–854. 17503341 10.14670/HH-22.847

[17] Mor G (2008) Inflammation and pregnancy: the role of toll-like receptors in trophoblast-immune interaction. Ann N Y Acad Sci 1127: 121–128. 18443339 10.1196/annals.1434.006

[18] Gu L, Tseng S, Horner RM, Tam C, Loda M, et al (2000) Control of TH2 polarization by the chemokine monocyte chemoattractant protein-1. Nature 404: 407–411. 10746730 10.1038/35006097

[19] Ren G, Zhao X, Zhang L, Zhang J, L'Huillier A, et al (2010) Inflammatory cytokine-induced intercellular adhesion molecule-1 and vascular cell adhesion molecule-1 in mesenchymal stem cells are critical for immunosuppression. J Immunol 184: 2321–2328. 20130212 10.4049/jimmunol.0902023 PMC2881946

[20] Fukushima A, Kajiya H, Izumi T, Shigeyama C, Okabe K, et al (2010) Pro-inflammatory cytokines induce suppressor of cytokine signaling-3 in human periodontal ligament cells. J Endod 36: 1004–1008. 20478455 10.1016/j.joen.2010.02.027

[21] Fushimi S, Ogino T, Hara J, Takahata T, Wakabayashi H, et al (2009) Forced expression of suppressor of cytokine signaling 3 in T cells protects the development of concanavalin A-induced hepatitis in mice. Clin Immunol 133: 437–446. 19766538 10.1016/j.clim.2009.08.015

[22] Jones RL, Hannan NJ, Kaitu'u TJ, Zhang J, Salamonsen LA (2004) Identification of chemokines important for leukocyte recruitment to the human endometrium at the times of embryo implantation and menstruation. J Clin Endocrinol Metab 89: 6155–6167. 15579772 10.1210/jc.2004-0507

[23] Carlino C, Stabile H, Morrone S, Bulla R, Soriani A, et al (2008) Recruitment of circulating NK cells through decidual tissues: a possible mechanism controlling NK cell accumulation in the uterus during early pregnancy. Blood 111: 3108–3115. 18187664 10.1182/blood-2007-08-105965

[24] Keefe D, Shi L, Feske S, Massol R, Navarro F, et al (2005) Perforin triggers a plasma membrane-repair response that facilitates CTL induction of apoptosis. Immunity 23: 249–262. 16169498 10.1016/j.immuni.2005.08.001

[25] Wang L, Sun R, Li P, Han Y, Xiong P, et al (2011) Perforin is recaptured by natural killer cells following target cells stimulation for cytotoxicity. Cell Biol Int 10.1042/CBI20110242 21981014

[26] Abrahams VM, Visintin I, Aldo PB, Guller S, Romero R, et al (2005) A role for TLRs in the regulation of immune cell migration by first trimester trophoblast cells. J Immunol 175: 8096–8104. 16339547 10.4049/jimmunol.175.12.8096

[27] Fest S, Aldo PB, Abrahams VM, Visintin I, Alvero A, et al (2007) Trophoblast-macrophage interactions: a regulatory network for the protection of pregnancy. Am J Reprod Immunol 57: 55–66. 17156192 10.1111/j.1600-0897.2006.00446.x

[28] Kim YM, Romero R, Chaiworapongsa T, Kim GJ, Kim MR, et al (2004) Toll-like receptor-2 and -4 in the chorioamniotic membranes in spontaneous labor at term and in preterm parturition that are associated with chorioamnionitis. Am J Obstet Gynecol 191: 1346–1355. 15507964 10.1016/j.ajog.2004.07.009

[29] Kumazaki K, Nakayama M, Yanagihara I, Suehara N, Wada Y (2004) Immunohistochemical distribution of Toll-like receptor 4 in term and preterm human placentas from normal and complicated pregnancy including chorioamnionitis. Hum Pathol 35: 47–54. 14745724 10.1016/j.humpath.2003.08.027

[30] Adams KM, Lucas J, Kapur RP, Stevens AM (2007) LPS induces translocation of TLR4 in amniotic epithelium. Placenta 28: 477–481. 17055575 10.1016/j.placenta.2006.08.004 PMC3067058

[31] Rindsjo E, Holmlund U, Sverremark-Ekstrom E, Papadogiannakis N, Scheynius A (2007) Toll-like receptor-2 expression in normal and pathologic human placenta. Hum Pathol 38: 468–473. 17239937 10.1016/j.humpath.2006.09.009

[32] Proost P, Wuyts A, Van Damme J (1996) Human monocyte chemotactic proteins-2 and -3: structural and functional comparison with MCP-1. J Leukoc Biol 59: 67–74. 8558070 10.1002/jlb.59.1.67

[33] Gu L, Rutledge B, Fiorillo J, Ernst C, Grewal I, et al (1997) In vivo properties of monocyte chemoattractant protein-1. J Leukoc Biol 62: 577–580. 9365111 10.1002/jlb.62.5.577

[34] Karpus WJ, Kennedy KJ, Kunkel SL, Lukacs NW (1998) Monocyte chemotactic protein 1 regulates oral tolerance induction by inhibition of T helper cell 1-related cytokines. J Exp Med 187: 733–741. 9480983 10.1084/jem.187.5.733 PMC2212174

[35] Gonzalo JA, Lloyd CM, Wen D, Albar JP, Wells TN, et al (1998) The coordinated action of CC chemokines in the lung orchestrates allergic inflammation and airway hyperresponsiveness. J Exp Med 188: 157–167. 9653092 10.1084/jem.188.1.157 PMC2525544

[36] He YY, Du MR, Guo PF, He XJ, Zhou WH, et al (2007) Regulation of C-C motif chemokine ligand 2 and its receptor in human decidual stromal cells by pregnancy-associated hormones in early gestation. Hum Reprod 22: 2733–2742. 17704101 10.1093/humrep/dem208

[37] Allavena P, Bianchi G, Zhou D, van Damme J, Jilek P, et al (1994) Induction of natural killer cell migration by monocyte chemotactic protein-1, -2 and -3. Eur J Immunol 24: 3233–3236. 7805752 10.1002/eji.1830241249

[38] Nokihara H, Yanagawa H, Nishioka Y, Yano S, Mukaida N, et al (2000) Natural killer cell-dependent suppression of systemic spread of human lung adenocarcinoma cells by monocyte chemoattractant protein-1 gene transfection in severe combined immunodeficient mice. Cancer Res 60: 7002–7007. 11156403

[39] Yu CR, Ortaldo JR, Curiel RE, Young HA, Anderson SK, et al (1999) Role of a STAT binding site in the regulation of the human perforin promoter. J Immunol 162: 2785–2790. 10072525

[40] Zhang J, Scordi I, Smyth MJ, Lichtenheld MG (1999) Interleukin 2 receptor signaling regulates the perforin gene through signal transducer and activator of transcription (Stat)5 activation of two enhancers. J Exp Med 190: 1297–1308. 10544201 10.1084/jem.190.9.1297 PMC2195674

[41] Tsuboi N, Yoshikai Y, Matsuo S, Kikuchi T, Iwami K, et al (2002) Roles of toll-like receptors in C-C chemokine production by renal tubular epithelial cells. J Immunol 169: 2026–2033. 12165529 10.4049/jimmunol.169.4.2026

[42] Schaefer TM, Desouza K, Fahey JV, Beagley KW, Wira CR (2004) Toll-like receptor (TLR) expression and TLR-mediated cytokine/chemokine production by human uterine epithelial cells. Immunology 112: 428–436. 15196211 10.1111/j.1365-2567.2004.01898.x PMC1782499

[43] Koga K, Mor G (2010) Toll-like receptors at the maternal-fetal interface in normal pregnancy and pregnancy disorders. Am J Reprod Immunol 63: 587–600. 20367625 10.1111/j.1600-0897.2010.00848.x PMC3025804

[44] Yu XW, Zhang XW, Li X (2009) Soluble tumor necrosis factor receptor mediates cell proliferation on lipopolysaccharide-stimulated cultured human decidual stromal cells. Int J Mol Sci 10: 2010–2018. 19564935 10.3390/ijms10052010 PMC2695263

[45] Puhr M, Santer FR, Neuwirt H, Susani M, Nemeth JA, et al (2009) Down-regulation of suppressor of cytokine signaling-3 causes prostate cancer cell death through activation of the extrinsic and intrinsic apoptosis pathways. Cancer Res 69: 7375–7384. 19738059 10.1158/0008-5472.CAN-09-0806

[46] Liu J, Lu H, Huang R, Lin D, Wu X, et al (2005) Peroxisome proliferator activated receptor-gamma ligands induced cell growth inhibition and its influence on matrix metalloproteinase activity in human myeloid leukemia cells. Cancer Chemother Pharmacol 56: 400–408. 15838654 10.1007/s00280-005-1029-9

[47] Krampera M, Cosmi L, Angeli R, Pasini A, Liotta F, et al (2006) Role for interferon-gamma in the immunomodulatory activity of human bone marrow mesenchymal stem cells. Stem Cells 24: 386–398. 16123384 10.1634/stemcells.2005-0008

[48] Braunschweig A, Poehlmann TG, Busch S, Schleussner E, Markert UR (2011) Signal transducer and activator of transcription 3 (STAT3) and Suppressor of Cytokine Signaling (SOCS3) balance controls cytotoxicity and IL-10 expression in decidual-like natural killer cell line NK-92. Am J Reprod Immunol 66: 329–335. 21385272 10.1111/j.1600-0897.2011.00989.x

