# Supplementary figures and images for: Monocyte Chemoattractant Protein-1 Secreted by Decidual Stromal Cells Inhibits NK Cells Cytotoxicity by Up-Regulating Expression of SOCS3
Source: PLoS One. 2012 Jul 27;7(7):e41869. doi: 10.1371/journal.pone.0041869 (PMC3407114; doi:10.1371/journal.pone.0041869)

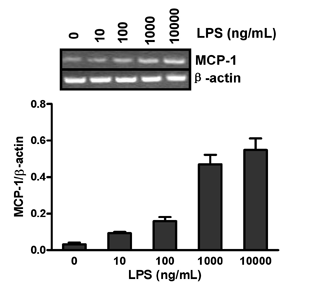

Supplement: Figure S1 — LPS stimulates MCP-1 mRNA expression in DSCs in a dose-dependent fashion. DSCs cells (5×10^5^ cells/mL) were seeded onto 6-well plates, and incubated with LPS of different concentration (10 ng/mL, 100 ng/mL, 1 µg/mL, 10 µg/mL) for 24 hours. The mRNA expression was detected by RT-PCR. The image shown is representative of 3 independent experiments and the graph represents the quantitation of the bands using β-actin as an internal standard. The data are shown as the mean ± SD (n = 3). (TIF) [file pone.0041869.s001.tif]
